# Predictive Value of Electrocardiographic Markers Versus Echocardiographic and Clinical Measures for Appropriate ICD Shocks in Heart Failure Patients

**DOI:** 10.3390/jcm14155506

**Published:** 2025-08-05

**Authors:** Özkan Bekler, Süleyman Diren Kazan, Hazar Harbalioğlu, Onur Kaypakli

**Affiliations:** 1 Department of Cardiology, Istanbul Medipol University, Istanbul 31001, Turkey; 2 Department of Cardiology, Hatay Mustafa Kemal University, Hatay 31060, Turkey; diren.kazan@hotmail.com (S.D.K.); onurkaypakli@hotmail.com (O.K.); 3 Department of Cardiology, Adana City Hospital, Adana 01170, Turkey; hazarhmail@hotmail.com

**Keywords:** Electrocardiography, ICD shock, QRS-T angle, QTc, Tp-e/QT ratio, heart failure

## Abstract

**Background:** Despite the survival benefit of ICDs in patients with HFrEF, most recipients do not receive appropriate therapy during follow-up. Existing risk models based on echocardiographic and clinical parameters show limited predictive accuracy for arrhythmic events. This study aimed to assess whether ECG-derived markers outperform conventional measures in predicting appropriate ICD shocks. **Methods:** This retrospective observational study included 375 patients with HFrEF who underwent ICD implantation for primary prevention at least six months before study enrollment. Twelve-lead surface ECGs were analyzed for a QTc interval, Tp-e/QT ratio, frontal QRS-T angle, and maximum deflection index (MDI). Clinical, echocardiographic, and arrhythmic event data obtained from device interrogations were evaluated. Receiver operating characteristic (ROC) curve analysis and multivariate logistic regression were performed to identify independent predictors of appropriate ICD shocks. **Results:** Patients who experienced appropriate ICD shocks had significantly higher rates of a complete bundle branch block, digoxin use, QRS duration, QTc, Tp-e/QT ratio, frontal QRS-T angle, MDI, and right-ventricular pacing ratio. Conversely, beta-blocker use was significantly lower in this group. In multivariate analysis, independent predictors of appropriate shocks included the patient’s digoxin use (OR = 2.931, *p* = 0.003), beta-blocker use (OR = 0.275, *p* = 0.002), frontal QRS-T angle (OR = 1.009, *p* < 0.001), QTc interval (OR = 1.020, *p* < 0.001), and Tp-e/QT ratio (OR = 4.882, *p* = 0.050). The frontal QRS-T angle had a cutoff value of 105.5° for predicting appropriate ICD shocks (sensitivity: 73.6%, specificity: 85.2%, AUC = 0.758, *p* < 0.001). **Conclusions:** Electrocardiographic markers, particularly the frontal QRS-T angle, QTc interval, and Tp-e/QT ratio, demonstrated superior predictive power for appropriate ICD shocks compared to conventional echocardiographic and clinical measures. These easily obtainable, non-invasive ECG parameters may improve current risk stratification models and support more individualized ICD implantation strategies.

## 1. Introduction

Heart failure (HF) is a prevalent and serious condition, affecting 1–2% of adults in developed countries and contributing significantly to cardiovascular morbidity and mortality [1]. Sudden cardiac death (SCD), often caused by malignant ventricular arrhythmias, accounts for a major portion of these deaths [2]. Implantable cardioverter-defibrillators (ICDs) are the primary preventive strategy for SCD in patients with HF and reduced ejection fraction (HFrEF) [3,4].

Although ICDs reduce mortality by terminating life-threatening ventricular arrhythmias, real-world data reveal that the majority of patients implanted for primary prevention never receive appropriate therapy during follow-up [5]. Despite evidence from landmark trials such as MADIT-II and SCD-HeFT, current selection criteria, primarily based on left-ventricular ejection fraction (LVEF) and the NYHA functional class, remain suboptimal in predicting arrhythmic risk [6,7]. LVEF is widely used to assess systolic function, but does not reflect the electrical instability underlying malignant arrhythmias. Indeed, some patients with reduced LVEF never experience ICD shocks, while others with preserved systolic function may still succumb to sudden cardiac death [8]. These paradoxes emphasize the limitations of structural and symptom-based scoring systems and highlight the need for more accurate, mechanism-based risk stratification strategies. Additionally, considering the potential complications of ICD therapy, including inappropriate shocks, device-related infections, and psychosocial burden, improving patient selection remains a critical priority [9,10].

Electrocardiography (ECG), a widely available, non-invasive modality, offers valuable insights into electrical instability. Parameters such as the frontal QRS-T angle, QTc interval, Tp-e interval, and Tp-e/QT ratio have been linked to ventricular arrhythmogenesis [11]. Others, like fragmented QRS, reflect myocardial scarring and carry prognostic value in cardiomyopathies [12]. These ECG markers are thought to capture abnormalities in depolarization and repolarization, including electrical dispersion and heterogeneity, which are key pathophysiological substrates of malignant ventricular arrhythmias. Accordingly, they may offer incremental value in predicting appropriate ICD shocks, especially in patients with inconclusive structural parameters. However, the comparative predictive performance of these markers versus echocardiographic and clinical variables remains underexplored [13].

Compared to echocardiographic markers, which primarily reflect structural and functional myocardial remodeling, ECG-derived markers provide direct insight into the heart’s electrophysiological properties. Echocardiography remains essential for assessing left-ventricular function, wall-motion abnormalities, and chamber dimensions; however, it may fail to detect micro-level electrical instability [14]. ECG markers, in contrast, are simple, reproducible, cost-effective, and easily obtainable at bedside without specialized imaging equipment. Their limitations include variability due to heart rate, autonomic influences, and technical artifacts. Given their complementary nature, the combined use of ECG and echocardiographic data may enhance risk stratification and optimize patient selection for ICD implantation.

This study aimed to evaluate the predictive value of baseline ECG parameters in forecasting appropriate ICD shocks in HFrEF patients and to compare their performance against traditional clinical and echocardiographic indicators.

## 2. Materials and Methods

### 2.1. Study Design and Population

This retrospective observational study was conducted at Hatay Mustafa Kemal University Research Hospital between January 2023 and January 2025. Patients with HFrEF (LVEF ≤ 40%) who underwent ICD implantation for primary prevention at least six months before enrollment and were under regular follow-up were included. Exclusion criteria were paced ventricular rhythms, a complete bundle branch block, congenital heart disease, secondary prevention ICD indication, and poor-quality ECGs. Clinical, echocardiographic, and arrhythmic event data were retrieved from medical records.

### 2.2. Device Interrogation

ICD interrogations were performed during routine visits using manufacturer-specific programmers. Stored ventricular arrhythmic events—including appropriate ICD shocks, the number of shocks, non-sustained VT (NSVT), monitored VT, ATP-terminated VT, and the right-ventricular pacing (RV pacing) percentage—were analyzed. Supraventricular arrhythmias, artifacts, and inappropriate shocks were excluded. Two experienced electrophysiologists independently reviewed all episodes using intracardiac electrograms and device algorithms.

### 2.3. Echocardiographic Assessment

Transthoracic echocardiography (TTE) was performed for all patients within 1 week prior to ICD implantation. LVEF was assessed using the biplane Simpson’s method, in accordance with the American Society of Echocardiography recommendations [15]. All echocardiographic measurements were manually performed by cardiology specialists who were blinded to patients’ clinical outcomes and ICD therapy status. Standard 2D imaging was used, and additional parameters such as left-ventricular dimensions and wall-motion abnormalities were also evaluated when available.

### 2.4. Electrocardiographic Parameters

Standard 12-lead ECGs (25 mm/s, 10 mm/mV) recorded within three months of interrogation were analyzed. The frontal QRS-T angle was calculated as the absolute difference between QRS and T axes, adjusted to a maximum of 180° [16]. The maximum deflection index (MDI) was defined as the ratio of QRS duration to RR interval [17]. QT interval was measured from the Q wave onset to the T wave end, and QTc was calculated using Bazett’s formula [18]. The Tp-e interval and Tp-e/QT ratio were assessed using the tangent method in precordial leads (preferably V5 or V6) [11]. All ECG measurements were performed manually by two blinded cardiologists; discrepancies of >5% were resolved by consensus.

### 2.5. Statistical Analysis

Data were analyzed using SPSS v21.0 (SPSS Inc., Chicago, IL, USA). Continuous variables were reported as the mean ± standard deviation (SD) or median with interquartile range (IQR), depending on the normality of distribution assessed via the Kolmogorov–Smirnov test. Categorical variables were presented as frequencies and percentages. Comparisons were made using the independent *t*-test or Mann–Whitney U test for continuous variables and chi-square or Fisher’s exact test for categorical variables. Receiver operating characteristic (ROC) curve analysis was used to determine optimal cutoff values. ROC curve analysis was conducted for selected ECG markers (QTc, Tp-e/QT, frontal QRS-T angle, MDI) based on their clinical relevance and prior evidence of association with ventricular arrhythmias. Variables with *p* < 0.05 in univariate analysis were entered into multivariate logistic regression. A *p*-value < 0.05 was considered statistically significant.

## 3. Results

### 3.1. Clinical and Laboratory Parameters

Baseline clinical and laboratory characteristics are summarized in Table 1. Digoxin use, presence of non-sustained VT, monitored VT, and VT terminated with ATP were significantly more common among patients who experienced appropriate ICD shocks (all *p* < 0.001). In contrast, beta-blocker use was significantly lower in this group (*p* = 0.033).

### 3.2. Electrocardiographic and Echocardiographic Parameters

As shown in Table 2, patients with appropriate shocks had a significantly higher QRS duration, QTc interval, Tp-e/QT ratio, frontal QRS-T angle, maximum deflection index (MDI), RV pacing ratio, and a greater prevalence of complete bundle branch block (all *p* < 0.05). Echocardiographic parameters such as LVEF, LV dimensions, and LA size did not differ significantly between groups.

### 3.3. ROC Curve Analysis

**Figure 1:**
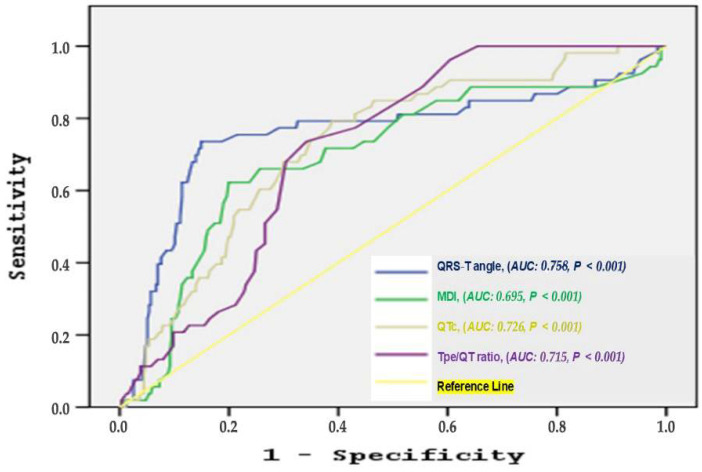
ROC curve analysis to determine predictive value of QTc, Tpe/QT ratio, frontal QRS-T angle and maximum deflection index for the occurrence of appropriate shock.

ROC curve analysis was conducted to evaluate the predictive performance of electrocardiographic parameters (Figure 1). The frontal QRS-T angle yielded a cutoff value of 105.5°, with a sensitivity of 73.6% and a specificity of 85.2% (AUC = 0.758, *p* < 0.001). For the QTc interval, the optimal cutoff was 443.5 ms, demonstrating a sensitivity of 79.2% and specificity of 61.1% (AUC = 0.726, *p* < 0.001). The Tp-e/QT ratio showed a cutoff value of 0.205, with 73.6% sensitivity and 65.5% specificity (AUC = 0.715, *p* < 0.001). The maximum deflection index (MDI) had a cutoff of 0.405, with 71.7% sensitivity and 61.9% specificity (AUC = 0.691, *p* < 0.001).

### 3.4. Multivariate Logistic Regression Analysis

Multivariate binary logistic regression analysis (Table 3) identified digoxin use (OR = 2.931, *p* = 0.003), beta-blocker use (OR = 0.275, *p* = 0.002), frontal QRS-T angle (OR = 1.009 per degree, *p* < 0.001), QTc interval (OR = 1.020 per millisecond, *p* < 0.001), and the Tp-e/QT ratio (OR = 4.882, *p* = 0.050) as independent predictors of appropriate ICD shocks. Notably, each 10° increase in the frontal QRS-T angle was associated with a 9% increase in the likelihood of experiencing an appropriate shock. Other parameters, including MDI, the RV pacing ratio, QRS duration, and the presence of a complete bundle branch block, did not remain statistically significant in the multivariate model (*p* > 0.05).

Among the ECG parameters, patients who experienced appropriate ICD shocks exhibited significantly prolonged QTc intervals, elevated Tp-e/QT ratios, and wider frontal QRS-T angles compared to those without shocks. These findings suggest increased ventricular repolarization dispersion and electrical heterogeneity in this group, which are known substrates for malignant ventricular arrhythmias. Additionally, a higher maximum deflection index (MDI) and greater QRS duration were also observed, indicating impaired conduction and delayed depolarization, both of which may contribute to arrhythmic vulnerability.

## 4. Discussion

In this retrospective study, we assessed the predictive value of selected surface ECG parameters, specifically the frontal QRS-T angle, QTc interval, Tp-e/QT ratio, and MDI, in identifying patients with HFrEF at risk of receiving appropriate ICD shocks. Our findings indicate that these electrocardiographic markers outperformed conventional echocardiographic and clinical variables in predicting life-threatening ventricular arrhythmias. Among the parameters evaluated, the frontal QRS-T angle demonstrated the highest predictive accuracy (AUC = 0.758), followed by the QTc interval (AUC = 0.726), Tp-e/QT ratio (AUC = 0.715), and MDI (AUC = 0.695), with all metrics achieving statistical significance (*p* < 0.001).

Our findings align with a growing body of evidence supporting the utility of surface ECG as a practical and effective tool for arrhythmic risk stratification. Among the evaluated markers, the frontal QRS-T angle emerged as the strongest independent predictor for appropriate ICD therapy. Physiologically, this parameter reflects the spatial deviation between ventricular depolarization and repolarization vectors. A widened QRS-T angle indicates increased global electrical heterogeneity within the myocardium, which facilitates the development of reentrant circuits and predisposes to malignant arrhythmias such as VT and VF. Several prior studies have similarly identified the QRS-T angle as a powerful prognostic marker, independently associated with sudden cardiac death and appropriate ICD interventions [19,20,21,22]. This widening is often linked to underlying structural abnormalities, including myocardial fibrosis, transmural scarring, or electrical dyssynchrony between myocardial regions such as the septum and lateral wall [23,24]. Experimental models have further shown that alterations in the QRS-T angle reflect regional repolarization disparities and vector disorganization [25], which disrupt electrical homogeneity and promote the formation of arrhythmogenic zones [26]. Clinically, this simple, non-invasive ECG parameter may reveal latent electrophysiological risk profiles not evident in structural imaging alone [27].

Another important observation in our study was the significant association between prolonged QTc intervals and appropriate ICD shocks. QTc prolongation is commonly linked to delayed ventricular repolarization, which may arise from structural heart disease such as myocardial ischemia or fibrosis, or from ion channel dysfunction. Both mechanisms extend the duration of the ventricular action potential and enhance the risk of early afterdepolarizations, which can trigger polymorphic ventricular tachyarrhythmias, including torsades de pointes [28,29,30]. Although QTc is known to be influenced by factors such as heart rate, electrolyte disturbances, and medication use, our findings demonstrated a robust association between prolonged QTc and appropriate ICD therapies, independent of these confounders. Prior studies have similarly reported that every 10-millisecond increase in QTc is associated with a significantly higher risk of sudden cardiac death and arrhythmic events, supporting the clinical relevance of QTc as a predictive marker in diverse cardiac populations [28,31].

The Tp-e interval and Tp-e/QT ratio are electrocardiographic markers that reflect the transmural dispersion of repolarization—the difference in repolarization timing among endocardial cells, M cells, and epicardial cells. M cells are characterized by the longest action potential duration, and the Tp-e interval represents the time between the peak of the T wave (corresponding to epicardial repolarization) and its end (representing M cell repolarization). Prolongation of the Tp-e interval facilitates phase two reentry, a well-established mechanism underlying malignant ventricular arrhythmias [32]. In our study, higher Tp-e/QT ratios were significantly associated with appropriate ICD shocks, emphasizing their value as indicators of electroanatomical vulnerability. Experimental evidence has demonstrated that an increased Tp-e duration enhances transmural dispersion, thereby promoting both monomorphic and polymorphic VT [33]. The seminal work by Antzelevitch and Sicouri first described the arrhythmogenic potential of delayed M cell repolarization, forming the basis for the clinical application of Tp-e-based indices. Notably, the Tp-e/QT ratio has been validated as a strong and independent predictor of ICD therapy in patients undergoing primary prevention [34,35].

The MDI, defined as the ratio of the QRS duration to RR interval, offers insight into ventricular conduction dynamics by quantifying depolarization time in relation to the cardiac cycle length [17]. Elevated MDI values are indicative of delayed intraventricular conduction, which may arise from diffuse myocardial fibrosis, dysfunction of the His-Purkinje system, or localized conduction slowing. These conduction disturbances promote unidirectional blocks and facilitate reentrant circuit formations, thereby increasing the risk of monomorphic ventricular tachycardia [36]. As a non-invasive, surface ECG-derived metric, MDI serves as a potential surrogate for identifying conduction delays and may reduce the need for invasive electrophysiological testing in select cases. Although less extensively studied than other repolarization markers, MDI and similar indices have been associated with sustained ventricular arrhythmias in prior investigations [17,36,37,38]. In our study, elevated MDI values were significantly associated with appropriate ICD shocks in the univariate analysis; however, this association did not persist in the multivariate model. Despite not being an independent predictor, MDI was included in the ROC analysis due to its univariate significance and the support from previous studies suggesting its mechanistic relevance to arrhythmogenic risk.

Despite its long-standing role as the primary criterion for ICD implantation, LVEF has notable limitations in predicting arrhythmic events [36]. Although randomized trials have demonstrated survival benefits of ICDs in patients with reduced LVEF, real-world data reveal that a substantial proportion, up to two-thirds, never experience appropriate therapy during follow-up [7,39,40,41]. Consistent with previous studies, including the EU-CERT-ICD trial, our findings showed no significant association between echocardiographic parameters and appropriate shocks, underscoring the limited predictive value of structural metrics [42]. LVEF reflects systolic function but fails to capture key electrophysiological disturbances such as electrical instability, repolarization dispersion, and conduction delay [43,44]. Moreover, sudden cardiac death can occur even in the absence of overt structural heart disease, and emerging data suggest that surface ECG markers may outperform LVEF in mortality prediction [2,45].

Similarly, standard clinical variables such as age, NYHA class, and comorbidity burden are known to influence overall prognosis but often fail to predict arrhythmic risk [46,47]. In our study, these clinical factors were not associated with appropriate ICD therapies, reinforcing prior evidence regarding their limited discriminatory power.

While LVEF remains a cornerstone in current ICD implantation guidelines, its prognostic limitations in arrhythmia prediction are increasingly evident. Studies consistently show that a majority of patients with low LVEF do not experience appropriate ICD therapy, while many sudden cardiac deaths occur in individuals with preserved LVEF. This underscores a disconnect between mechanical dysfunction and arrhythmogenic risk. ECG-derived markers offer a direct window into myocardial electrical vulnerability. Among these, the frontal QRS-T angle reflects global dispersion of repolarization and is strongly associated with myocardial fibrosis and increased arrhythmic risk, as corroborated by imaging-validated studies that link a widened QRS-T angle with scar burdens and conduction heterogeneity [48]. Similarly, the Tp-e interval and Tp-e/QT ratio serve as robust indicators of transmural repolarization dispersion, a well-documented precursor to reentrant ventricular arrhythmias [32]. These parameters outperform conventional markers such as the NYHA class and comorbidity scores, which reflect overall heart failure severity but not arrhythmic potential. Additionally, markers like the MDI, although not always independently predictive, signal conduction slowing and have been associated with ventricular tachyarrhythmias in univariate models. The combined use of ECG markers offers superior sensitivity and arrhythmia-specific risk profiling, as supported by multicentric analyses [42,49]. Given their cost-effectiveness, ease of acquisition, and mechanistic relevance, these non-invasive tools are emerging as essential adjuncts and potentially alternatives to echocardiographic and clinical parameters in guiding ICD therapy in HFrEF populations.

In contrast, ECG-derived parameters, particularly a QRS-T angle > 120°, a Tp-e/QT ratio > 0.25, and a QTc > 470 ms, exhibited superior sensitivity in identifying patients at risk. Given their accessibility, reproducibility, and low cost, these markers may be especially valuable for risk stratification in patients with preserved or mid-range LVEF who are currently not eligible for ICD therapy under existing guidelines. Future prospective multicenter studies are warranted to confirm these findings and support their integration into clinical decision-making.

## 5. Study Limitations and Future Implications

This study has several limitations. First, its retrospective and single-center design may limit the generalizability of the results. Second, although ECG parameters were manually measured by two blinded cardiologists, interobserver variability remains a potential source of bias. Third, we did not incorporate advanced imaging techniques (e.g., cardiac MRI) or biomarkers (e.g., NT-proBNP), which could have enhanced the characterization of myocardial substrate and arrhythmic risk. Fourth, the lack of an external validation cohort limits the generalizability and external applicability of our findings. Future multicenter prospective studies with external validation are needed to confirm these results. Another limitation is the imbalance in group sizes between patients with and without appropriate ICD shocks. This discrepancy may affect statistical power and model calibration, potentially limiting the robustness of comparative analyses. However, this reflects the real-world prevalence of arrhythmic events in primary prevention ICD populations and underscores the need for larger multicenter datasets.

Future studies with larger, multicenter cohorts and external validation are needed to confirm our findings and establish standardized cutoff values for ECG markers. The integration of ECG-derived parameters with existing clinical and imaging-based risk models may enhance stratification accuracy for ICD therapy. Additionally, combining these markers with emerging technologies such as artificial intelligence or machine learning may enable real-time, individualized risk assessment in clinical practice.

## 6. Conclusions

Surface ECG-derived parameters, including the QRS-T angle, QTc interval, Tp-e/QT ratio, and maximum deflection index, demonstrated stronger predictive value for appropriate ICD therapy than traditional echocardiographic or clinical variables. These findings highlight the potential role of ECG-based markers in refining current risk stratification models and advancing toward more individualized, evidence-based ICD implantation strategies.

## Figures and Tables

**Table 1:** Comparison of baseline clinical and laboratory parameters in patients with and without appropriate shock.

	Patients Without Appropriate Shock n = 322	Patients with Appropriate Shock n = 53	*p*
Age (years)	63.0 ± 12.6	63.8 ± 13.8	0.665
Gender (Male, %)	250 (77.6)	45 (84.9)	0.231
Hypertension (n, %)	181 (56.2)	25 (47.2)	0.220
DM (n, %)	144 (44.7)	21 (39.6)	0.488
Type of CMP (ischemic/dilated)	243/79	42/11	0.551
Type of ICD (VVIR/DDDR)	233/89	44/9	0.102
Aspirin (n, %)	202 (62.7)	32 (60.4)	0.743
Digoxin (n, %)	66 (20.5)	22 (41.5)	0.001
Beta Blocker (n, %)	265 (82.3)	37 (69.8)	0.033
Amiodarone (n, %)	31 (9.6)	5 (9.4)	0.965
Nondihydropyridine CCB (n, %)	2 (0.6)	1 (1.9)	0.368
ACEi/ARB (n, %)	237 (73.6)	38 (71.7)	0.771
Sprinolactone (n, %)	220 (68.3)	31 (58.5)	0.159
Statin (n, %)	180 (55.9)	28 (52.8)	0.677
Hemoglobin (g/dl)	12.8 ± 2.1	12.8 ± 2.0	0.977
White blood cell (×10^9^/L)	9.7 ± 3.7	10.4 ± 4.0	0.732
Creatinine (mg/dL)	1.20 ± 0.69	1.22 ± 0.63	0.756
LDL cholesterol (mg/dL)	96.0 ± 38.1	96.6 ± 35.1	0.929
Triglycerides (mg/dL)	155.3 ± 90.4	161.3 ± 115.2	0.936
VT terminated with ATP (n, %)	31 (9.6)	44 (83.0)	<0.001
Monitor VT (n, %)	47 (14.6)	29 (54.7)	<0.001
Non-sustained VT (n, %)	74 (23.0)	35 (66.0)	<0.001

Abbreviations: DM, diabetes mellitus; CMP, cardiomyopathy; ICD, implantable cardioverter-defibrillator; VVIR, ventricular inhibited rate-responsive pacing; DDDR, dual-chamber rate-responsive pacing; VT, ventricular tachycardia; ATP, antitachycardia pacing; ACEi, angiotensin-converting enzyme inhibitor; ARB, angiotensin receptor blocker; CCB, calcium channel blocker; LDL, low-density lipoprotein.

**Table 2:** Comparison of the baseline electrocardiographic and echocardiographic features of the study population.

Variable	Patients Without Appropriate Shock n = 322	Patients with Appropriate Shock n = 53	*p*
QRS duration (ms)	107.7 ± 23.5	117.4 ± 26.1	0.007
QTc (ms)	433.43 ± 40.9	461.1 ± 29.7	<0.001
Tpe/QT ratio	0.20 ± 0.16	0.26 ± 0.09	<0.001
Frontal QRS-T angle (degree)	−8.0 ± 111.4	85.4 ± 118.4	<0.001
Maximum deflection index	0.49 ± 0.65	0.58 ± 0.33	<0.001
Complete BBB (n, %)	35 (10.9)	13 (24.5)	0.006
Atrial Fibrillation (n, %)	40 (12.4)	8 (15.1)	0.590
RV pacing ratio	3.4 ± 10.7	7.4 ± 15.8	0.007
Days after implantation (days)	756.7 ± 561.4	773.7 ± 680.5	0.741
LV end-diastolic diameter (mm)	62.3 ± 7.9	61.5 ± 7.4	0.497
LV end-systolic diameter (mm)	51.3 ± 7.9	50.4 ± 7.7	0.351
LVEF (%)	29.7 ± 10.0	30.5 ± 10.5	0.732
LA end-diastolic diameter (mm)	40.4 ± 9.8	40.9 ± 9.0	0.846

Abbreviations: QTc, corrected QT interval; Tp-e, T-peak to T-end interval; RV, right ventricle; LV, left ventricle; LVEF, left-ventricular ejection fraction; LA, left atrium; BBB, bundle branch block; ms, milliseconds; mm, millimeters.

**Table 3:** Multivariate binary logistic regression analyses to determine the independent predictors of the occurrence of appropriate shock.

Variable	*p*	Odds Ratio	95% CI	
			Lower	Upper
Digoxin use	0.003	2.931	1.441	5.964
Beta bloker use	0.002	0.275	0.120	0.635
Frontal QRS-T angle	<0.001	1.009	1.006	1.012
QTc	<0.001	1.020	1.011	1.030
Tpe/QT ratio	0.050	4.882	1.003	23.769
Maximum deflection index	0.344			
RV pacing ratio	0.656			
QRS duration	0.934			
Complete BBB	0.545			

Abbreviations: QTc, corrected QT interval; Tp-e, T-peak to T-end interval; RV, right ventricle; BBB, bundle branch block; OR, odds ratio; CI, confidence interval.

## Data Availability

The data supporting the findings of this study are available from the corresponding author upon reasonable request.
